# Longitudinal Association Between Physical Activity and School Bullying in Adolescents: A Cross-Lagged Panel Model

**DOI:** 10.3390/bs15091236

**Published:** 2025-09-11

**Authors:** Kanglin Wang, Fen Qiu

**Affiliations:** School of Physical Education, Wuhan University of Technology, Wuhan 430070, China

**Keywords:** physical activity, school bullying, cross-lagged panel model, longitudinal study, gender differences, adolescents

## Abstract

Background: School bullying represents a critical global public health issue among adolescents. Although existing evidence suggests physical activity (PA) may reduce bullying risk, longitudinal data on bidirectional associations and gender-specific variations remain limited. This study employed a cross-lagged panel model (CLPM) to investigate the temporal dynamics between PA and school bullying (SB) and examine gender-moderating effects. Methods: A cohort of 577 middle school students (294 boys, 283 girls; mean age = 14.31 ± 2.1 years) from seven schools across Wuhan, Shijiazhuang, and Chengdu completed three-wave longitudinal assessments over 9 months (September 2024–March 2025). Validated instruments included the School Bullying Scale (SBS) and Physical Activity Rating Scale (PARS). The CLPM analysis evaluated bidirectional predictive pathways, with gender-stratified multi-group comparisons. Results: Significant bidirectional negative associations emerged: (1) PA at T1/T2 predicted reduced SB at T2/T3 (*β* = −0.14 to −0.26, *p* < 0.001). (2) SB at T1/T2 predicted decreased PA at T2/T3 (*β* = −0.27 to −0.38, *p* < 0.001). (3) Gender significantly moderated these relationships, with PA conferring stronger protective effects against subsequent SB in males (*β* = −0.35 vs. −0.21 for PA→SB paths). Conversely, SB triggered earlier and more pronounced PA reductions in males (*β* = −0.42 vs. −0.29 for SB→PA paths). Conclusions: PA and SB demonstrate stable bidirectional negative associations in adolescents, with significant gender divergence. Males exhibit greater resilience to bullying through PA engagement but heightened vulnerability to PA reduction post-victimization. These findings underscore PA-based interventions as promising bullying mitigation strategies, necessitating gender-tailored implementation approaches.

## 1. Introduction

School bullying represents a significant global public health concern (Gore et al., 2011), with particularly high prevalence among children and adolescents (Swearer et al., 2012). Global epidemiological data indicate that approximately one-third of students experience bullying (UNESCO, 2017), corroborated by the Global School-based Student Health Survey (2003–2015) reporting a 30.5% prevalence rate among adolescents aged 12–17 years (Biswas et al., 2020). School bullying (SB) refers to the situation where students repeatedly or for a long time suffer harm in aspects such as physical, verbal, and interpersonal relationships on campus, resulting in physical or psychological trauma (Olweus, 1994). It can be divided into physical bullying, psychological bullying, relational bullying, and cyberbullying (Sterzing et al., 2020). This phenomenon fundamentally represents aggressive behavior characterized by power imbalance (Kallianta et al., 2021). Empirical evidence identifies disabled students, those with obesity, academic underachievers, and rural populations as particularly vulnerable subgroups (Hong et al., 2018). Victimization experiences detrimentally impact adolescents’ academic performance (Shetgiri, 2017), mental health (Han et al., 2025), and value development (Yin et al., 2024), with longitudinal research confirming persistent negative effects extending into adulthood, including compromised psychological functioning and impaired social competence (Y. Zhou et al., 2025).

### 1.1. Physical Activity and School Bullying

Physical activity (PA) constitutes a key intervention for promoting adolescent mental health development, with substantial empirical evidence confirming its significant benefits for emotional regulation and social adaptation (Sibold et al., 2015). These benefits are theorized to operate through multiple mechanisms, including neurophysiological pathways (e.g., endorphin release and reduced cortisol) that improve mood and stress resilience, and psychosocial pathways that provide opportunities for mastery, social connection, and the development of cooperative skills. However, it is crucial to note that these positive outcomes are not automatic; they are heavily dependent on the social context in which PA occurs (Eslinger et al., 2021). Specifically, regular PA engagement not only effectively mitigates adolescent anxiety but also enhances self-regulation capacities through neurophysiological mechanisms (Zhao et al., 2020). In structured settings like physical education (PE), the teacher’s approach, the design of activities (e.g., emphasizing cooperation over pure competition), and the overall pedagogical intentions are paramount in maximizing these benefits and fostering an inclusive environment (MacPhail et al., 2023). Conversely, without careful design, PE can also be a context where vulnerabilities are exposed—through public performance, body image pressures, or highly competitive dynamics—potentially fostering exclusion or bullying (Jesina et al., 2022). Longitudinal studies demonstrate that adolescents engaging in PA ≥4 times weekly exhibit significantly reduced aggressive behavior incidence (Méndez et al., 2019), while Waasdorp et al. (2019) further established a significant negative correlation between PA participation and bullying perpetration (Waasdorp et al., 2019). This protective effect can be understood through a social–ecological lens (Griffin & Gross, 2004), wherein PA, particularly team sports, provides a structured environment for social practice (Shialos, 2021) that fosters peer bonding, cultivates rule awareness, and enhances social skills through embodied cognition processes (Oh & Yoo, 2023). By building social capital and self-efficacy, PA may reduce perceived vulnerability, thereby mitigating the risk of victimization for adolescents.

Notably, Nikolaou and Crispin (2022) observed that although competitive sports participants display marginally higher bullying perpetration rates, their victimization risk approaches zero (Nikolaou & Crispin, 2022). This suggests that the social context and competence in PA are crucial; adolescents with lower physical competence or body image concerns may paradoxically be at higher risk of victimization in PA settings (e.g., gym classes and sports), as they might be perceived as “easy targets” (Gualdi-Russo et al., 2022), aligning with the “provocative victim” subtype associated with motor skill limitations (Griffin & Gross, 2004). From a social–ecological perspective, school physical education (PE) curricula provide structured environments for social practice that are uniquely rich in functional diversity (e.g., hedonic, agonistic, communicative, and expressive) (Brown et al., 2014; Rocliffe et al., 2024). It is precisely through this diversity of activities—from cooperative games to competitive team sports—that PE can explicitly foster cooperation, inclusion, and value-based learning by creating immediate, embodied experiences of shared goals, rule-following, and mutual respect. Team sports specifically facilitate peer bonding while cultivating rule awareness and cooperative models that enhance social skills through embodied cognition processes (Peng et al., 2025a; Sawka et al., 2014).

Conversely, a distinct psychological process explains the reverse pathway. Bullying victimization is consistently associated with a dual behavioral pattern: reduced physical activity coupled with increased sedentary behavior (Pacífico et al., 2024). This can be conceptualized through a trauma-avoidance model (Orcutt et al., 2020), whereby victims develop avoidance behaviors—such as skipping gym classes or avoiding school grounds—to evade bullies and re-traumatization. This maladaptive profile potentially exacerbates bullying-related health risks through activity insufficiency. Empirical evidence indicates that victims aged 17–20 demonstrate 40% higher incidence rates of persistent anxiety and social phobia compared to non-victims, substantially diminishing sports participation motivation (Dubey et al., 2022). Neurophysiological research further reveals that bullying experiences alter functional connectivity between the posterior cingulate cortex and default mode network, inducing anhedonia and vitality reduction that predisposes victims to fatigue during routine activities (D’Urso et al., 2024). This creates a negative feedback loop: victimization leads to PA avoidance, which reduces opportunities for building the very social and physical competencies that protect against bullying, potentially perpetuating victim status. This potential for a self-reinforcing cycle aligns with Olweus’ conceptualization of bullying as a stable phenomenon where victimization can become entrenched over time (Olweus, 1994). Particularly vulnerable populations include students with disabilities, who face 2–3 times greater bullying risk due to motor skill limitations (L. Yang, 2021). Students with physical functional impairments or social skill deficits face a higher rate of exclusion when participating in group sports, which in turn leads to a significant decrease in their physical activity participation.

### 1.2. Gender Differences and Current Research

Significant gender disparities characterize bullying dynamics. Females exhibit substantially lower victimization rates during physical education classes compared to males (Alfonso-Rosa et al., 2020), while males demonstrate both higher perpetration prevalence (Obregon-Cuesta et al., 2022) and increased vulnerability to victimization (M. Yang et al., 2022). Cross-cultural evidence further confirms gender-based variations in bullying typologies and intervention attitudes, as demonstrated in Spanish adolescents (Piñeiro et al., 2022). These disparities extend beyond prevalence to the very manifestation of bullying. Extensive research indicates a clear typological divergence: males typically report higher rates of physical and verbal bullying perpetration, while females are more likely to engage in and experience relational bullying (e.g., social exclusion and rumor-spreading) (Gomes et al., 2022; Li & Liu, 2025; Provenzano & Volk, 2021; Voisin et al., 2023). This divergence is theorized to stem from gendered socialization practices that often encourage overt aggression and dominance in boys, while emphasizing relational intimacy and social network management in girls (Leaper & Farkas, 2015). Furthermore, motivational differences exist; male bullying often aligns with instrumental goals and establishing dominance hierarchies, whereas female bullying is more frequently reactive and relationship-oriented (Reijntjes et al., 2013). In terms of victimization, males demonstrate increased vulnerability to physical victimization, potentially exacerbated by norms condoning physical aggression in male peer groups and higher participation in contact sports (Sacks et al., 2003). Females, conversely, may be more susceptible to psychological and relational victimization (Sun et al., 2025). These typological differences underscore the critical necessity of examining bullying dynamics through a gendered lens, as the use of aggregate measures may obscure these critical patterns in how bullying is enacted and experienced.

### 1.3. Present Study and Hypotheses

Current research on physical activity–bullying relationships predominantly utilizes cross-sectional methodologies (Garcia et al., 2021; Liu et al., 2024; Q. Zhang & Deng, 2024), which inherently constrain causal inference and temporal sequencing analysis (X. Wang & Cheng, 2020). Guided by the integrated social–ecological and psychological avoidance framework, this study employs a longitudinal design to examine the bidirectional relationship between physical activity (PA) and school bullying (SB) victimization and to test whether these pathways are moderated by gender. The following hypotheses are proposed:

**H1:**  *PA and SB will exhibit significant negative bidirectional associations over time.*

**H1a:**  *PA will negatively predict subsequent SB.*

**H1b:**  *SB will negatively predict subsequent PA.*

**H2:**  *gender will moderate these associations.*

## 2. Method

### 2.1. Participants

This study employed a three-wave, 9-month longitudinal design to address the limitations of cross-sectional research and examine the bidirectional associations between PA and SB using cross-lagged panel modeling. The moderating role of gender was also investigated. This prospective cohort study employed a 9-month longitudinal design (September 2024 to March 2025) with three-wave data collection. Using cluster sampling, we recruited participants from seven middle schools across Wuhan, Shijiazhuang, and Chengdu. Before questionnaire administration, written informed consent was obtained from all participants and legal guardians following a detailed explanation of study protocols. The Ethics Review Committee of Wuhan University of Technology approved the protocol, with strict adherence to the Declaration of Helsinki ethical standards.

On-site survey procedures were standardized across all three assessments. After implementing systematic exclusion criteria—including school transfers (*n* = 3), substantial non-response (>20% missing items, *n* = 11), and irregular response patterns (e.g., straight-lining, *n* = 23)—the final analytic sample comprised 577 adolescents (294 males, 283 females; mean age = 14.31 ± 2.1 years). Attrition analysis via independent samples *t*-tests revealed no significant baseline differences (T1) between retained and attrited participants regarding gender distribution (*p* = 0.75), physical activity levels (*p* = 0.48), or bullying scores (*p* = 0.81). Little’s MCAR test confirmed data missingness was random (*χ*^2^/*df* = 1.18, *p* = 0.18), supporting the use of full-information maximum likelihood estimation (Enders, 2010).

### 2.2. Research Tools

#### 2.2.1. Physical Activity Rating Scale (PARS)

The PARS, a validated scale revised by Liang (Liang, 1994), was employed to assess the PA levels of middle school students and investigate their exercise habits. This scale was originally published in the *Chinese Mental Health Journal* (Liang, 1994) and has been widely used in adolescent studies. This scale evaluates PA intensity, duration, and frequency, with each dimension rated on a 5-point scale from 1 (low) to 5 (high). The amount of PA is calculated using the formula PA  =  Intensity × Duration × Frequency. The activity levels are categorized as follows: 0–19 points indicate low activity, 20–42 points indicate moderate activity, and 43–100 points indicate high activity. In this study, the internal consistency coefficients of the three measurements were 0.84, 0.83, and 0.81, respectively.

#### 2.2.2. School Bullying Scale (SBS)

The bullying subscale was adapted from the Olweus Bullying Questionnaire (Olweus, 1997) and validated in Chinese adolescents by W. Zhang and Wu (1999), consisting of 12 items. Six items assess bullying perpetration (e.g., “I hit, kicked, or pushed other students”), and six items assess victimization (e.g., “Other students left me out of things on purpose”). The scale captures a spectrum of behaviors including physical, verbal, and relational bullying (X. Chen et al., 2022). Higher scores indicated a higher level of bullying perpetration or victimization (M. Yang et al., 2022). It uses a 5-point Likert scale to measure the frequency of bullying behaviors. Six items assess bullying, and six items assess victimization. The frequency of occurrence is rated from 0 to 5, ranging from “never happened” to “several times a week”. In this study, the internal consistency coefficients of the three measurements were 0.94, 0.93, and 0.90, respectively.

### 2.3. Data Processing and Analysis

Missing value analysis, reliability test, common method bias test, descriptive statistics, and correlation analysis were carried out using SPSS 25.0 and Mplus 8.3 software, and the longitudinal invariance test of SBS was conducted (Little & Card, 2013). Since the time intervals are the same, a cross-lagged model can be directly established, and the chi-square test is used to evaluate the goodness of fit and adaptability. A value of *p* < 0.05 indicates a significant difference. Due to the large sample size, we further introduce the Standardized Root Mean Square Residual (SRMR) < 0.05, TLI, and CFI > 0.90 to evaluate the model fit (Baribeau et al., 2022; Kline, 2016).

## 3. Results

### 3.1. Common Method Bias Test

The SBS in this study adopted the self-report of the subjects, which may have a certain degree of common method bias. Therefore, Harman’s single-factor analysis was used to analyze the SBS. The results showed that at the three time points of T1, T2, and T3, the number of factors with eigenvalues greater than 1 was 4, and the variance explained by the first factor was 29.29%, 24.89%, and 19.70% respectively, all of which were less than the critical standard of 40% (H. Zhou & Long, 2004). Therefore, there is no serious common method bias in this study.

### 3.2. Correlation Analysis

Bivariate correlation analyses between physical activity and school bullying outcomes across three time points (T1, T2, and T3) revealed statistically significant negative associations at all measurement waves (r = −0.35 to −0.52, *p* < 0.001; Table 1), demonstrating robust temporal stability in effect directionality and magnitude that substantiates the application of longitudinal modeling techniques.

### 3.3. Longitudinal Measurement Invariance

Table 2 presents the longitudinal measurement invariance test results for the School Bullying Scale (SBS) conducted via three-stage progressive constraints (Little & Card, 2013). Configural invariance demonstrated excellent model fit (*χ*^2^/*df* = 2.38, CFI = 0.956, SRMR = 0.041), satisfying conventional thresholds (*χ*^2^/*df* < 3, CFI > 0.95, SRMR < 0.05). Metric invariance met cross-lagged modeling requirements (ΔCFI = 0.003 < 0.01), while scalar invariance was not fully supported (ΔCFI = 0.009 > 0.01). Given this study’s focus on structural relationships rather than latent mean comparisons, metric invariance provides a sufficient psychometric foundation for subsequent analyses, consistent with established methodological guidelines (Cheung & Rensvold, 2002; Vandenberg & Lance, 2000).

The results of the longitudinal measurement invariance test of the SBS are shown in Table 2 below. The test follows the three-stage progressive constraint method. The results show that the morphological equivalence model fits well (*χ*^2^/*df* = 2.38 < 3, CFI = 0.956 > 0.95, SRMR = 0.041 < 0.05). Metric invariance meets the analysis conditions of the cross-lagged model (ΔCFI = 0.003 < 0.01). Strong equivalence is not fully established (ΔCFI = 0.009), but since this study focuses on the relationships between variables rather than mean changes, weak equivalence is sufficient to support subsequent analyses (F. F. Chen, 2007).

### 3.4. Cross-Lagged Model Analysis

**Figure 1:**
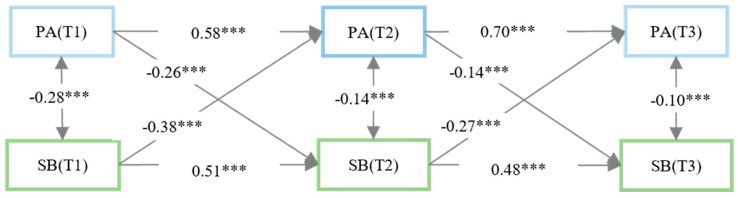
Diagram of the cross-lagged model (standardized coefficient). Significance: PA = physical activity, SB = school bullying, *** *p* < 0.001.

The three-wave cross-lagged panel model (Figure 1) examined bidirectional temporal relationships between physical activity and school bullying. Model fit indices demonstrated excellent data correspondence, satisfying conventional thresholds: *χ*^2^/*df* = 7.54 (*p* < 0.001), CFI = 0.984, TLI = 0.920, SRMR = 0.038, and RMSEA = 0.042 (Hu & Bentler, 1999). The results showed that physical activity and school bullying were significantly autoregressive and negatively correlated at T1, T2, and T3 (*p* < 0.001). Crucially, bidirectional predictive pathways were confirmed: (1) PA at T1/T2 negatively predicted SB at T2/T3 (*β* = −0.14 to −0.26, *p* < 0.001); (2) SB at T1/T2 negatively predicted PA at T2/T3 (*β* = −0.27 to −0.38, *p* < 0.001).

### 3.5. Multiple Group Comparisons

Multi-group structural equation modeling tested gender’s moderating role in the PA-SB dynamic. Measurement invariance was established through sequential constraints: non-significant differences between configural (*χ*^2^(184) = 402.57, CFI = 0.960, SRMR = 0.041) and metric invariance models (*χ*^2^(196) = 410.32, CFI = 0.958, SRMR = 0.044; Δ*χ*^2^(12) = 7.75, *p* = 0.805; ΔCFI= −0.002) confirmed factor loading equivalence across genders (Little & Card, 2013).

The stepwise constraint of cross-lagged paths revealed significant model deterioration when equating PA→SB trajectories across genders (Δ*χ*^2^(4) = 13.86, *p* < 0.001, ΔCFI = −0.007), with gender-stratified coefficients demonstrating significantly stronger protective effects of PA against subsequent SB in males: PA (T1) → SB (T2) (*β_male_* = −0.35 vs. *β_female_* = −0.21; Δχ^2^(1) = 6.94, *p* < 0.001); and PA (T2) → SB (T3) (*β_male_* = −0.32 vs. *β_female_* = −0.19; Δ*χ*^2^(1) = 5.83, *p* < 0.001). Constraining SB → PA paths yielded non-significant global deterioration (*p* > 0.05), yet males exhibited heightened early vulnerability in SB (T1) → PA (T2) *(β_male_* = −0.42 vs. *β_female_* = −0.29; Δ*χ*^2^(1) = 3.74, *p* = 0.053), while SB (T2) → PA (T3) showed no gender difference (*β* = −0.15 vs. −0.03; Δ*χ*^2^(1) = 0.32, *p* = 0.21), indicating temporally specific amplification of bullying’s physical activity suppression in males during initial phases.

### 3.6. Exploratory Analysis by Bullying Type and Gender

Given the documented gender differences in bullying manifestations, we conducted exploratory analyses examining the prevalence of different bullying types (physical, verbal, and relational) by gender at T1. Consistent with previous literature (X. Wang & Cheng, 2020), chi-square tests revealed significant gender differences (*p* < 0.001). Boys reported significantly higher rates of physical perpetration and victimization, while girls reported higher rates of relational perpetration and victimization. Verbal bullying did not differ significantly by gender. The specific results are shown in Table 3.

Furthermore, multi-group CLPM analyses were run separately for physical and relational victimization scores. The protective effect of PA was significant across types but was strongest in reducing physical victimization for boys (β = −0.38, *p* < 0.001) and relational victimization for girls (β = −0.24, *p* < 0.01).

## 4. Discussion

This study confirms the cross-temporal stability of physical activity (PA) and school bullying (SB), aligning with Olweus’ bullying cycle theory positing self-reinforcing behavioral persistence (Olweus, 1994). This temporal consistency likely stems from adolescents’ relatively stable contextual environments, where PA patterns demonstrate limited short-term variability (Cheng & Yu, 2022).

### 4.1. Bidirectional Association Between Physical Activity and School Bullying

Our findings support a bidirectional negative relationship between PA and SB, consistent with the social–ecological and avoidance-based theoretical frameworks introduced earlier. The protective effect of PA aligns with its role in enhancing social capital and self-efficacy, thereby reducing perceived vulnerability. Conversely, the pathway from SB to reduced PA is consistent with avoidance behavior, where victims withdraw from contexts where bullying occurs. This is consistent with H1, H1a, and H1b.

Consistent with prior evidence (Garcia et al., 2021; Méndez et al., 2019), optimal behavioral outcomes emerge when exercise duration is maintained ≤1 h at moderate intensity, significantly attenuating adolescent aggression (Yu et al., 2024). The primary physiological pathway involves enhanced physical capacity, reducing vulnerability to “weakness-triggered” bullying; regular PA mitigates physique-targeted aggression through improved motor competence and body composition (Torchyan et al., 2022). Crucially, whereas social isolation constitutes a core bullying risk factor (Newman et al., 2005), PA provides regulated social contexts that counteract the controversial “exposure-aggression” paradigm (den Hamer & Konijn, 2015). Team sports specifically foster cooperative experiences (e.g., basketball passing and football tactics) that strengthen social identity and interpersonal trust (Lubans et al., 2016; K. Wang & Qiu, 2025).

Conversely, bullying victimization precipitates PA reduction primarily through social-avoidance-mediated behavioral withdrawal (Stirling et al., 2011), reaffirming the established SB-PA deficiency association (García-Hermoso et al., 2020; Van Vliet, 2010). This may escalate into compensatory behaviors like electronic device addiction (Ding & Zhang, 2022; Peng et al., 2025b), initiating a self-perpetuating cycle: physiologically, PA deficiency impairs dopaminergic function, exacerbating depressive symptomatology (Sampasa-Kanyinga et al., 2020); psychologically, low mood further suppresses exercise motivation, engendering negative self-schemata. Without timely intervention, this bidirectional reinforcement loop may crystallize maladaptive lifestyles with lifelong health consequences (Blanchflower & Bryson, 2024).

### 4.2. Gender-Specific Pathways

The gendered patterns observed further reflect social-ecological influences. Males’ stronger protective benefit from PA may stem from the higher social status and peer validation associated with athletic success in male peer groups (Lawler et al., 2022), consistent with social capital theory. Conversely, males’ sharper decline in PA post-victimization may reflect a greater threat to athletic identity and stronger adherence to norms discouraging perceived weakness (Arnocky & Vaillancourt, 2014), triggering stronger avoidance responses. This is consistent with H2.

Further analysis reveals significant gender-based asymmetric vulnerability in the PA-SB bidirectional pathways (Hankin et al., 2007): males derive substantially stronger protective benefits from PA against bullying compared to females. This divergence stems from males’ greater propensity to build social capital through high-intensity team sports (e.g., basketball and football) (Bang et al., 2024), establishing support networks that mitigate victimization (Andersson et al., 2024). This process is heavily influenced by sociocultural norms that often valorize high-intensity, competitive team sports as a masculine domain, thereby amplifying the social status and protective peer alliances boys derive from excelling in these gender-normative activities. The competitive–cooperative dynamics inherent in such activities foster group identity and status hierarchies, thereby reducing bullying risk. Conversely, the trauma-avoidance cycle (Van Vliet, 2010) manifests more acutely in males, where post-victimization PA reduction is both immediate and pronounced due to gendered behavioral paradigms—bullying dismantles the “physical strength” identity integral to male athletic participation, inducing the dual impairment of exercise motivation and social confidence (Bruner et al., 2022). Females exhibit superior psychosocial resilience when confronting bullying, demonstrating enhanced emotional stability and relationship maintenance (Bedrov & Gable, 2023; Peleg & Peleg, 2025), which partially attenuates bullying’s negative impact on PA engagement. This likely stems from girls’ bullying being more relational than PA-focused (Demol et al., 2022). Thus, PA may protect females primarily by building general psychological resources (like emotional regulation and social support) that foster cross-context resilience, explaining its significant but smaller protective effect (Eslinger et al., 2021; Kelly et al., 2012).

Our exploratory findings further illuminate these gendered pathways of the replication of established patterns—higher physical bullying among boys and relational bullying among girls (J. Wang et al., 2009). The multi-group analysis by bullying type suggests that the protective effect of PA may be most potent against the form of bullying most salient to each gender: reducing physical victimization risks for boys and mitigating relational victimization for girls. This nuanced finding extends our main analysis by demonstrating that PA’s benefits are not monolithic but are channeled through gendered social ecologies and bullying contexts.

## 5. Limitations and Future Prospects

This study acknowledges primary limitations: (1) Although physical activity (PA) assessment predominantly utilized validated questionnaires, inherent self-reporting biases may persist; future research should incorporate objective monitoring via accelerometry or wearable devices. (2) The undifferentiated treatment of bullying perpetration and victimization dimensions obscures potential differential associations with PA; subsequent analyses will employ multidimensional bullying frameworks. (3) Geographical sampling restricted to select Chinese provinces constrains national representativeness, necessitating cross-cultural examinations of bullying–PA dynamics. (4) While the cross-lagged panel model conflates between- and within-person effects—a recognized methodological constraint—its application remains appropriate for capturing developmental dynamics in this adolescent cohort. (5). Despite using validated scales, self-reported data are susceptible to biases such as social desirability. Furthermore, variability in PE contexts across the seven schools (e.g., teacher approach and activities) may have influenced the results, a factor not controlled for in this study.

## 6. Conclusions

This study confirms that adolescents’ physical activity (PA) and school bullying (SB) exhibit cross-temporal stability and reciprocal negative prediction, with gender-asymmetric vulnerability: males show stronger PA-mediated protection but greater post-victimization PA reduction. Schools and parents must implement PA-based anti-bullying interventions with gender-tailored strategies—leveraging team sports for males and resilience building for females—while providing trauma-informed psychological support.

## Figures and Tables

**Table 1:** Correlation analysis between variables.

Variables	1	2	3	4	5	6
1 PA (T1)	1					
2 PA (T2)	0.68 ***	1				
3 PA (T3)	0.61 ***	0.73 ***	1			
4 SB (T1)	−0.42 ***	−0.38 ***	−0.35 ***	1		
5 SB (T2)	−0.39 ***	−0.46 ***	−0.41 ***	0.67 ***	1	
6 SB (T3)	−0.36 ***	−0.44 ***	−0.52 ***	0.59 ***	0.71 ***	1
M	25.3	26.1	27.4	15.7	14.8	13.5
SD	8.2	7.9	8.5	5.3	4.8	4.1

Significance: PA = physical activity, SB = school bullying, *** *p* < 0.001.

**Table 2:** Results of the longitudinal invariance test.

		*χ*^2^/*df*	SRMR	CFI	|ΔSRMR|	|ΔCFI|
	configural	2.38	0.041	0.956		
SB	metric	2.29	0.043	0.953	0.002	0.003
	scalar	2.25	0.046	0.947	0.003	0.009

**Table 3:** Prevalence of bullying types by gender at time 1.

Type	Role	Boys (%)	Girls (%)	*χ* ^2^	*p*
Physical	Perpetrator	15.3	5.7	15.2	<0.001
Physical	Victim	18.0	8.1	12.8	<0.001
Relational	Perpetrator	10.2	16.3	5.1	0.024
Relational	Victim	12.9	19.4	4.9	0.027
Verbal	Perpetrator	22.1	19.1	0.9	0.342
Verbal	Victim	25.5	23.0	0.5	0.468

## Data Availability

The data supporting this study’s findings are available from the corresponding author upon reasonable request.
